# EKG-Beurteilung im Rettungs- und Notarztdienst in Deutschland: Ergebnisse einer Querschnittsstudie

**DOI:** 10.1007/s00063-025-01252-1

**Published:** 2025-02-25

**Authors:** Angela Gerhard, Yvonne Treusch, Luis Möckel, Karin Kohlstedt, Thomas Hofmann

**Affiliations:** 1 Fachbereich Gesundheit und Soziales, HSD Hochschule Döpfer (University of Applied Sciences), Potsdam, Deutschland; 2 Deutsche Gesellschaft für Rettungswissenschaften e. V., Aachen, Deutschland; 3 Institut für Forschung in der Operativen Medizin (IFOM), Universität Witten/Herdecke, Neufelder Str. 34, 51067 Köln, Deutschland

**Keywords:** EKG-Interpretation, EKG-Ausbildung, EKG-Fortbildung, Rettungsdienst, Notfallsanitäter, ECG interpretation, ECG education, ECG advanced training, Emergency medical services, Paramedics

## Abstract

**Hintergrund:**

Die Registrierung eines 12-Kanal-Elektrokardiogramms (EKG) stellt einen unverzichtbaren Teil der Basisdiagnostik im Rettungs- und Notarztdienst dar. Für die Erstversorgung und Stellung einer Verdachtsdiagnose müssen Notfallsanitäter*innen (NFS) und Notärzt*innen (NA) gute Kenntnisse in der Interpretation des EKG besitzen. Trotzdem zeigen einige Studien auf, dass die EKG-Interpretationsfähigkeit von NFS und NA verbesserungswürdig ist.

**Ziel der Arbeit:**

Das Ziel war es, die EKG-Interpretationskompetenz von NFS und NA in Deutschland zu ermitteln.

**Material und Methoden:**

Vom 22. Februar bis 22. März 2023 wurde eine Onlinebefragung durchgeführt. Es wurden Daten zu Geschlecht, Alter, Berufsausbildung, Berufserfahrung, Zeitpunkt der letzten EKG-Fortbildung und eine Selbsteinschätzung der EKG-Kompetenzen erhoben. Des Weiteren mussten 9 unterschiedliche EKG in Form von Single-Choice-Fragen interpretiert werden.

**Ergebnisse:**

Insgesamt 908 Befragte (NFS: 803; NA: 105) wurden in die Auswertung eingeschlossen, wovon 677 Teilnehmende alle EKG beantworteten. Im Durchschnitt schätzten alle 677 Teilnehmenden, die alle EKG beantwortet haben, die EKG zu 63,3 % korrekt ein (NFS: 61,4 %; NA: 76,5 %). Das EKG mit ST-Strecken-Hebungs-Myokardinfarkt (STEMI) in der Hinterwand wurde von 79,1 % (NFS: 78,1 %; NA: 86,7 %) der Befragten (*n* = 908) richtig eingeschätzt. Defizite bestanden bei der Interpretation von AV-Blöcken, Tachykardien, Vorhofflimmern, Schenkelblöcken, Schrittmacher-EKG und bei der Lagetypbestimmung. Die Selbsteinschätzung der Teilnehmenden bezüglich ihrer EKG-Kenntnisse korrelierte signifikant mit den tatsächlich erzielten Ergebnissen (*p* ≤ 0,001; *p*_*bonf*_ = 0,016; *ρ* = 0,378).

**Diskussion:**

Aufgrund von Defiziten in der Befundung von 12-Kanal-EKG sollten die EKG-Aus- und -Fortbildungsprogramme überarbeitet werden, um eine Qualitätssteigerung der Versorgung der Patient*innen in der prähospitalen Notfallmedizin zu bewirken.

**Zusatzmaterial online:**

Zusätzliche Informationen sind in der Online-Version dieses Artikels (10.1007/s00063-025-01252-1) enthalten.

## Abstract (en)

**Background:**

The recording of an electrocardiogram (ECG) is an essential part of basic diagnostics in the emergency medical services (EMS). To provide initial care and make a preliminary diagnosis, paramedics and emergency physicians (EP) need knowledge about the ECG interpretation. Nevertheless, a few studies show that knowledge of ECG interpretation of paramedics and emergency physicians must improve.

**Objectives:**

The aim of this study was to analyze the ECG interpretation skills among EMS staff and EP in Germany.

**Materials and methods:**

An online survey was conducted to assess these skills from 22 February to 22 March 2023. The survey collected details about gender, age, professional training, years of professional experience, time since the last ECG training, and self-rated ECG interpretation ability. Subsequently, 9 different ECG had to be interpreted in the form of single-choice questions.

**Results:**

In all, 908 participants (EMS: 803; EP: 105) were evaluated. The survey identified a knowledge deficit in the interpretation of ECG. On average, 63.3% (EMS: 61.4%; EP: 76.5%) of the ECG were interpreted correctly. The ECG with ST-elevation myocardial infarction (STEMI) of the posterior wall was identified correctly by 79.1% (EMS: 78.1%; EP: 86.7%) of the participants. Specific weak points in interpreting ECG were AV-blocks, tachycardias, atrial fibrillation, bundle branch blocks, pacemaker ECG and determining the ventricular axis. The participants’ self-assessment of their ECG skills correlated significantly with the results actually achieved (*p* ≤ 0.001; *p*_bonf_ = 0.016; *ρ* = 0.378).

**Conclusion:**

Based on the lack of diagnostic skills in the interpretation of a 12 lead ECG, continuous education programs should be revised to improve the quality of patient care in prehospital emergency medicine.

## Hintergrund und Fragestellung

Das 12-Kanal-EKG ist ein wichtiges, einfaches und kostengünstiges Diagnostikum im Rettungsdienst, das zur Erkennung von Erkrankungen des Herz-Kreislauf-Systems und zur Überwachung der Vitalzeichen von Patient*innen eingesetzt wird. Für die Erstversorgung und Stellung einer Verdachtsdiagnose müssen Notfallsanitäter*innen (NFS) und Notärzt*innen (NA) Kenntnisse in der korrekten Anlage und sicheren Interpretation des EKG besitzen. Darüber hinaus hat die schnelle und korrekte EKG-Interpretation einen großen Einfluss auf die weiteren Behandlungsmaßnahmen und damit auch auf die Prognose von Erkrankungsverläufen. Da das EKG häufig differenzialdiagnostisch wegweisend sein kann, sollte auch bei einem untypischen Beschwerdebild ein EKG zur Routine gehören [20]. Außerdem ist die Interpretation eines EKG für NFS Voraussetzung, um die Notfallsanitäteralgorithmen der Bundesländer und die der unterschiedlichen Rettungsdienstbereiche überhaupt anwenden zu können [17]. In der Untersuchung von Hofmann und Möckel [13] zur Beteiligung von NA bei Rettungsdiensteinsätzen in Deutschland zeigte sich, dass nur in 27,7 % der Rettungsdiensteinsätze überhaupt NA mit einem Notarzteinsatzfahrzeug involviert sind, sodass ausreichende EKG-Kenntnisse bei NFS unabdingbar sind. Israel [14] bezeichnet die EKG-Interpretation als eine Art Mustererkennung, die trainiert werden muss. Denn oft wird nur nach gezielten Kriterien analysiert, die die Anwender*innen kennen. EKG-Interpretationskompetenzen setzen sich aus einem umfangreichen Hintergrundwissen, disziplinierter Übung in der EKG-Interpretation und praktischer Erfahrung zusammen [15]. Es gibt nur wenige Studien, die die EKG-Beurteilungskompetenz von NFS und NA untersuchen. Diese Studien belegen, dass das Wissen zur EKG-Befundung verbesserungswürdig ist [3, 18, 19, 21]. Mencl et al. [18] führten eine Untersuchung mit 472 US-amerikanischen Paramedics in Ohio durch, bei der die Erkennung von ST-Strecken-Hebungs-Myokardinfarkten (STEMI) genauer betrachtet wurde. Die Sensitivität in der STEMI-Erkennung betrug 75 %. Auch Stockburger et al. [21] führten eine Studie zur EKG-Interpretation bei Myokardinfarkten und Rhythmusstörungen durch. Hierbei diagnostizierten NA in Berlin einen STEMI zu 62 % korrekt. Laut Ohlow et al. [19] erkannten 83 % der NA einen STEMI, allerdings wurden nur 30 % der Non-STEMI-EKG richtig befundet. Wie wichtig die adäquate EKG-Interpretation für die zügige Weiterbehandlung der Patient*innen ist, zeigt die Studie von Stockburger et al. [21]. Hier konnte bei prähospitaler STEMI-Diagnose ein beachtlicher Zeitgewinn erzielt werden.

Die primäre Fragestellung dieser Studie ist:

- Wie ist die Beurteilungskompetenz der EKG-Interpretation unter NFS und NA in Deutschland?

Weitere Fragestellungen sind:

- Gibt es Unterschiede in der EKG-Interpretation zwischen NFS und NA?
- Bestehen Unterschiede in der EKG-Interpretation in Abhängigkeit von der Berufserfahrung oder dem Zeitpunkt der letzten EKG-Fortbildung?

## Methoden

### Studiendesign und Untersuchungsmethoden

Bei dieser Studie handelt es sich um eine Querschnittstudie mit NFS und NA, die in der Zeit vom 22. Februar bis zum 22. März 2023 als Onlinebefragung mit SoSci Survey durchgeführt wurde (SoSci Survey GmbH, München 2023). Für die Rekrutierung der Teilnehmenden wurde ein Beitrag mit dem Link zur Umfrage in den sozialen Medien erstellt und durch die Autor*innen nach dem Schneeballverfahren verteilt. Zudem wurde der Beitrag über die Deutsche Gesellschaft für Rettungswissenschaften (DGRe) über die sozialen Medien geteilt. Teilnahmebedingungen für die Umfrage waren eine zum Zeitpunkt der Umfrage bestehende Tätigkeit als NFS oder NA in Deutschland und ein Mindestalter von 18 Jahren.

Die Erhebung erfolgte freiwillig und wurde im Rahmen des geltenden Datenschutzes durchgeführt. Ein Ethikantrag wurde bei der Ethikkommission der HSD Hochschule Döpfer, Köln, eingereicht und am 31.01.2023 bewilligt (Aktenzeichen: BEth_13_23). Die Berichterstattung erfolgte nach der STROBE-Leitlinie.

Der Fragebogen wurde speziell für diese Befragung entwickelt, da für die untersuchten Fragestellungen kein validierter Fragebogen existierte. Die Auswahl der zu beurteilenden EKG-Bilder wurde aufgrund der Aus- und Fortbildungsinhalte zum Thema EKG und der häufigsten sowie wichtigsten EKG getroffen [2, 4, 10, 19, 21]. Zur Überprüfung des Fragebogens auf fachliche Korrektheit wurden 2 Kardiologen als Experten hinzugezogen. Der Fragebogen bestand insgesamt aus 24 Fragen und beinhaltete Fragen zu soziodemografischen Daten wie Geschlecht, Alter, Bundesland (in dem die Tätigkeit ausgeübt wird) und Berufsausbildung bzw. Studium (Facharztausbildung). Daneben wurden die Berufserfahrung sowie die letzte Fortbildung im Bereich EKG abgefragt. Des Weiteren wurde gefragt, wie die Teilnehmenden selbst ihre EKG-Kenntnisse einschätzen und ob sie sich mehr Fortbildung zum Thema EKG wünschen. Anschließend folgten 9 EKG zur Beantwortung im Single-Choice-Format. Alle EKG des Fragebogens wurden einheitlich mit 50 mm/s Vorschub geschrieben. Die Teilnehmenden hatten bei jedem EKG die Möglichkeit, die Antwort „Ich kann das EKG nicht beurteilen“ zu wählen. Nachfolgende EKG wurden dargestellt, die genauen EKG-Bilder und Antwortmöglichkeiten sind dem Onlinesupplement zu entnehmen:

1. EKG mit STEMI über der Hinterwand;
2. EKG mit Bigeminus;
3. EKG mit AV-Block III°;
4. EKG mit ventrikulärer Tachykardie;
5. EKG mit supraventrikulärer Tachykardie;
6. EKG mit Schrittmacher in Betriebsart DDD;
7. EKG mit Tachyarrhythmia absoluta;
8. EKG mit Vorhofflimmern und Rechtsschenkelblock;
9. EKG mit Linkstyp.

### Datenauswertung

Für diese Studie wurde keine Stichprobengrößenberechnung durchgeführt, da ein Convenience Sampling verwendet wurde. Es wurde jedoch eine Stichprobengröße von *n* ≥ 800 Teilnehmenden (ca. 1 % aller Rettungsdienstmitarbeiter*innen in Deutschland) angestrebt. Mithilfe des Programms Statistical Package für Social Sciences (SPSS IBM, Böblingen, 2023) wurde die Analyse der Daten vorgenommen. Die Beurteilungskompetenz der NFS und NA wurde mithilfe des prozentualen Anteils der richtigen EKG-Einschätzungen operationalisiert. Für die Darstellung der Charakteristika der Studienteilnehmenden wurden Mittelwerte, Mediane, Interquartilsabstände (IQR), Standardabweichungen und prozentuale Anteile berechnet. Darüber hinaus wurden die Ergebnisse nach Berufserfahrung und Zeitpunkt der letzten EKG-Fortbildung analysiert. Zur Überprüfung der Unterschiede zwischen den verschiedenen Berufsgruppen wurde der Pearson‑χ^2^-Test und für die Überprüfung der Unterschiede zwischen der Berufserfahrung sowie dem Zeitpunkt der letzten EKG-Fortbildung der Mann-Whitney-U-Test verwendet. Im Anschluss wurde zwischen der Gesamtzahl der richtigen Single-Choice-EKG pro Gruppe und der Berufserfahrung sowie dem letzten Zeitpunkt einer EKG-Fortbildung die Spearman-Korrelation unter Berechnung von Rho ermittelt. Bei jeder Auswertung wurde die Gesamtzahl der Antworten angegeben, woraus sich die fehlenden Daten ableiten lassen. Als statistisch signifikant wurde bei allen Analysen ein *p*-Wert < 0,05 angesehen. Bei Analysen, die einen signifikanten *p*-Wert ergaben, wurde zusätzlich ein Bonferroni-korrigierter *p*-Wert berechnet (*p*_*bonf*_).

## Ergebnisse

### Charakteristika der Studienteilnehmenden

Die Befragung wurde von 2214 Personen angeklickt und von 1280 (57,8 %) Personen ausgefüllt. Eingeschlossen wurden alle Teilnehmenden, die mindestens das erste EKG bewertet haben, dies waren 908 Personen (70,9 %). Da keine Pflicht bestand, jede Frage zu beantworten, unterscheiden sich zum Teil die Anzahl der Teilnehmenden je Frage. Die Befragten (*n* = 907) waren zu 81,3 % männlich (*n* = 737), zu 18,3 % weiblich (*n* = 166) und zu 0,4 % divers (*n* = 4). Von den 908 Teilnehmenden waren 88,4 % NFS (*n* = 803) und 11,6 % NA (*n* = 105). Unter den NFS (*n* = 799) haben 36,5 % (*n* = 292) die 3‑jährige und 0,3 % (*n* = 2) die 5‑jährige Ausbildung absolviert. 40,2 % (*n* = 321) haben die Ergänzungsprüfung I (0–80 h Prüfungsvorbereitungskurs), 4,8 % (*n* = 38) die Ergänzungsprüfung II (480 h Kurs) und 2,1 % (*n* = 17) die Ergänzungsprüfung III (960 h Kurs) absolviert. Darüber hinaus haben 14,3 % (*n* = 114) die Vollprüfung durchlaufen und 1,9 % (*n* = 15) sind über eine sonstige Prüfung NFS geworden. Die 3 größten Gruppen der NA stellten mit 49,5 % Fachärzt*innen für Anästhesie (*n* = 52), 13,3 % Assistenzärzt*innen für Anästhesie (*n* = 14) und mit 12,4 % die Fachärzt*innen für Innere Medizin (*n* = 13) dar. Auf eine weitere Subgruppenanalyse der EKG nach Facharztrichtung wurde aber aufgrund dessen, dass die Ärzt*innen den Einsatz als Notärzt*innen und nicht als Fachvertreter*innen absolvieren, verzichtet. Das Alter der Befragten (*n* = 907) lag zwischen 19–70 Jahren mit einem Mittelwert von 35 ± 9 Jahre. Die Studie wurde mit Teilnehmenden aus allen 16 Bundesländern durchgeführt. Die Teilnehmenden (*n* = 907) waren am häufigsten mit 29,0 % (*n* = 263) 9–15 Jahre im Rettungsdienst in ihrer Position tätig. Tab. 1 zeigt die Charakteristika der Stichprobe.

**Tab. 1:** Charakteristika der Studienteilnehmenden

*Geschlecht*	Gesamt	NFS	NA
Frauen	18,3 % (*n* = 166)	17,8 % (*n* = 142)	22,9 % (*n* = 24)
Männer	81,3 % (*n* = 737)	82,0 %(*n* = 658)	75,2 % (*n* = 79)
Divers	0,4 % (*n* = 4)	0,2 % (*n* = 2)	1,9 % (*n* = 2)
*Alter*	MW: 35 ± 9 Jahre	MW:34 ± 9 Jahre	MW: 42 ± 9 Jahre
18–29 Jahre	34,3 % (*n* = 311)	38,8 % (*n* = 307)	3,8 % (*n* = 4)
30–44 Jahre	50,1 % (*n* = 454)	47,5 % (*n* = 385)	65,7 % (*n* = 69)
45–64 Jahre	15,4 % (*n* = 140)	13,7 % (*n* = 110)	28,6 % (*n* = 30)
> 64 Jahre	0,2 % (*n* = 2)	–	1,9 % (*n* = 2)
*Berufserfahrung (n* *=* *907)*			
< 1 Jahr	6,2 % (*n* = 56)	6,4 % (*n* = 50)	5,7 % (*n* = 6)
1–3 Jahre	16,0 % (*n* = 145)	15,9 % (*n* = 128)	16,2 % (*n* = 17)
4–8 Jahre	22,2 % (*n* = 202)	23,0 % (*n* = 185)	16,2 % (*n* = 17)
9–15 Jahre	29,0 % (*n* = 263)	29,1 % (*n* = 234)	27,7 % (*n* = 29)
16–20 Jahre	9,7 % (*n* = 88)	9,0 % (*n* = 72)	15,2 % (*n* = 16)
> 20 Jahre	16,9 % (*n* = 153)	16,6 % (*n* = 133)	19,0 % (*n* = 20)
*Zeitpunkt der letzten EKG-Fortbildung (n* *=* *906)*			
< 1 Monat	8,8 % (*n* = 80)	9,2 % (*n* = 74)	5,7 % (*n* = 6)
1–< 3 Monate	8,7 % (*n* = 79)	8,7 % (*n* = 70)	8,6 % (*n* = 9)
3–< 6 Monate	8,5 % (*n* = 77)	8,0 % (*n* = 64)	12,4 % (*n* = 13)
6 Monate–1 Jahr	24,4 % (*n* = 221)	25,8 % (*n* = 207)	13,3 % (*n* = 14)
> 1 Jahr	39,6 % (*n* = 359)	38,8 % (*n* = 311)	45,7 % (*n* = 48)
Bis jetzt keine EKG-Fortbildung besucht	9,9 % (*n* = 90)	9,4 % (*n* = 75)	14,3 % (*n* = 15)
*Berufsausbildung*			
Notfallsanitäter*innen (NFS)	88,4 % (*n* = 803)		
Notarzt/Notärztin (NA)	11,6 % (*n* = 105)		

### Teilnahme an EKG-Fortbildungen und EKG-Kompetenz-Selbsteinschätzung

Unter den Teilnehmenden (*n* = 906) haben 359 Befragte (39,6 %) die letzte EKG-Fortbildung vor mehr als einem Jahr besucht (NFS: 38,8 %, *n* = 311; NA: 45,7 %, *n* = 48) und 90 (9,9 %) haben noch nie an einer EKG-Fortbildung teilgenommen (NFS: 9,4 %, *n* = 75; NA: 14,3 %, *n* = 15). Allerdings wünschten sich 89,0 % (*n* = 807) mehr Fortbildung zum Thema EKG (NFS: 91,5 %, *n* = 734; NA: 69,5 %, *n* = 73).

Bei der Einschätzung der eigenen EKG-Kenntnisse, wobei ein Punkt keine bis sehr schlechte Kenntnisse und 5 Punkte sehr gute EKG-Kenntnisse bedeuteten, hatten alle Teilnehmenden einen Median von 3,0 (IQR 1). Unter den NFS lag der Median ebenfalls bei 3,0 (IQR 1). In dieser Berufsgruppe schätzten sich 1,6 % (*n* = 13) mit einem Punkt, 12,4 % (*n* = 99) mit 2 Punkten, 51,1 % (*n* = 409) mit 3 Punkten, 28,6 % (*n* = 229) mit 4 Punkten und 6,3 % (*n* = 50) mit 5 Punkten ein. Die Gruppe der NA schätzte sich im Median bei 4,0 (IQR 1) Punkten ein. 1 % (*n* = 1) der NA schätzte ihre EKG-Kenntnisse mit einem Punkt, 4,8 % (*n* = 5) mit 2 Punkten, 39,0 % (*n* = 41) mit 3 Punkten, 42,9 % (*n* = 45) mit 4 Punkten und 12,4 % (*n* = 13) mit 5 Punkten ein. Die Selbsteinschätzung der Teilnehmenden korrelierte signifikant mit den tatsächlich erzielten Ergebnissen (*p* ≤ 0,001; *p*_*bonf*_ = 0,016; *ρ* = 0,378). Teilnehmende, die ihre Fähigkeiten höher bewerteten, konnten auch mehr EKG richtig beantworten.

### Beurteilung der EKG

**Abb. 1:**
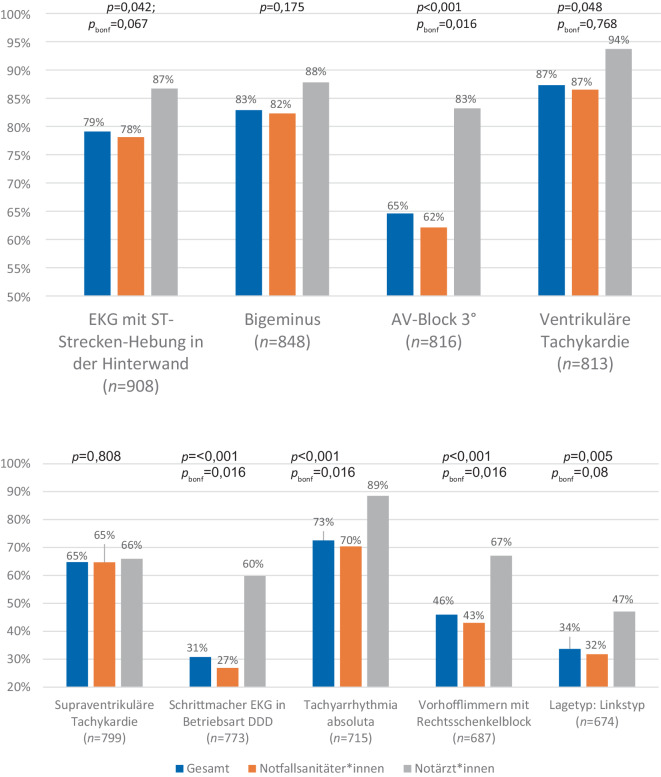
Auswertung der EKG-Befundung. Das Diagramm zeigt die Prozentzahl der richtigen EKG-Interpretationen je Gruppe. Die *p*-Werte zeigen die Unterschiede zwischen NFS und NA bei Durchführung eines χ^2^-Tests zur Prüfung der Signifikanz

EKG mit einer ST-Strecken-Hebung in der Hinterwand (NFS: 78,1 %; NA: 86,7 %; *p* = 0,042; *p*_*bonf*_ = 0,672), Überleitungsstörung im Sinne eines AV-Blocks III° (NFS: 62,1 %; NA: 83,2 %; *p* ≤ 0,001; *p*_*bonf*_ = 0,016), einem DDD-Schrittmacherrhythmus (NFS: 26,9 %; NA: 59,8 %; *p* ≤ 0,001; *p*_*bonf*_ = 0,016) oder ein EKG mit Vorhofflimmern und Rechtsschenkelblock (NFS: 43,0 %; NA: 67,1 %; *p* ≤ 0,001; *p*_*bonf*_ = 0,016) wurden von deutlich mehr NA im Vergleich zu NFS korrekt erkannt. Auch bei der Lagetypbestimmung wurde der Linkstyp von deutlich mehr NA im Vergleich zu NFS korrekt bestimmt (NFS: 31,8 %; NA: 47,1 %; *p* = 0,005; *p*_*bonf*_ = 0,08). Kein Unterschied bestand bei der Interpretation einer supraventrikulären Tachykardie (NFS: 64,7 %; NA: 66,0 %; *p* = 0,808). Eine allgemeine Auswertung der Häufigkeit der richtigen Antworten nach verschiedenen Berufsgruppen aller EKG befindet sich in Abb. 1.

**Abb. 2:**
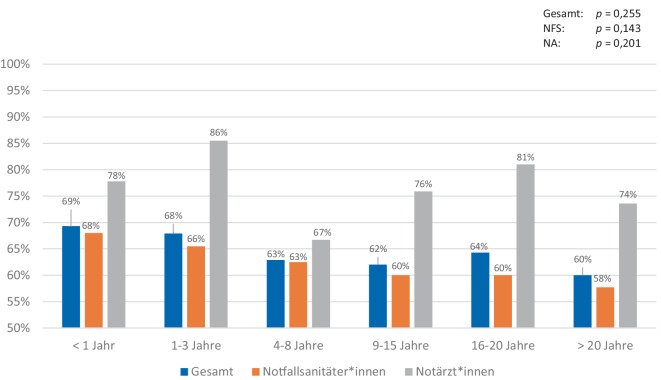
Häufigkeit richtiger EKG-Befundungen nach Berufserfahrung. Die *p*-Werte zeigen die Korrelationen zwischen der Berufserfahrung und der Häufigkeit der richtigen Antworten bei Durchführung von Spearman-Rho

**Abb. 3:**
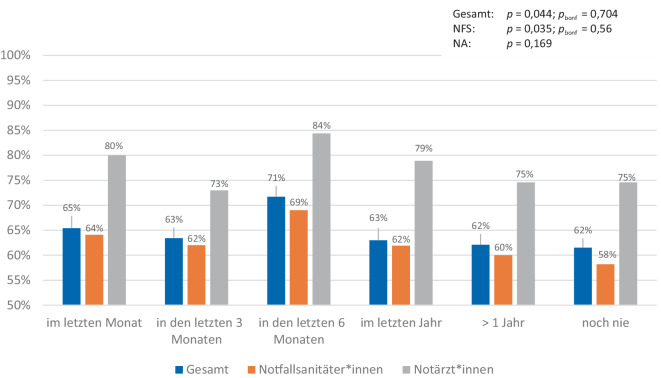
Häufigkeit richtiger EKG-Befundungen nach letzter EKG-Fortbildung. Die *p*-Werte zeigen die Korrelationen zwischen dem Zeitpunkt der letzten Fortbildung und der Häufigkeit der richtigen Antworten bei der Durchführung von Spearman-Rho

Insgesamt haben 677 Teilnehmende alle 9 Single-Choice-Fragen vollständig beantwortet und werden im Folgenden näher betrachtet. Im Mittel wurden die EKG zu 63,3 % (SD 23,3) sowie im Median zu 66,7 % (Min: 0 %; Max: 100 %) richtig beantwortet. NFS (*n* = 592) erreichten einen Mittelwert von 61,4 % (SD 23,3; Median: 66,7 %; Min: 0 %; Max: 100 %). NA (*n* = 85) schnitten mit einem Mittelwert von 76,5 % (SD 19,0; Median: 77,8 %; Min: 11,1 %; Max: 100 %) ab. Nach Berufserfahrung unterteilt (*n* = 674) haben die Teilnehmenden mit unter einem Jahr Berufserfahrung mit 69,3 % (NFS: 68,0 %; NA: 77,8 %) am besten bei der Befundung aller 9 Single-Choice-EKG abgeschnitten. Mit lediglich 60,0 % (NFS: 57,7 %; NA: 73,6 %) schnitten die Teilnehmenden mit mehr als 20 Jahren Berufserfahrung am schlechtesten ab. Im Bereich der Berufserfahrung zeigten sich unter den NFS (*p* = 0,143; *ρ* = −0,068) und unter den NA (*p* = 0,201; *ρ* = −0,148) keine Korrelation zwischen den Jahren an Berufserfahrung und der Anzahl der richtigen Antworten (siehe Abb. 2). Bei der Analyse der Häufigkeit der richtigen EKG-Befundungen und dem Zeitpunkt der letzten EKG-Fortbildung präsentierten sich Teilnehmende, die in den letzten 6 Monaten eine Fortbildung absolvierten, mit 71,7 % (NFS: 69,0 %; NA: 84,4 %) mit den besten Ergebnissen. Teilnehmende, die noch nie an einer EKG-Fortbildung teilgenommen haben, zeigten die wenigsten richtigen Antworten (61,5 % [NFS: 58,2 %; NA: 74,6 %]). Unter den NFS zeigte sich nach der Bonferroni-Korrektur (*p* = 0,035; *p*_*bonf*_ = 0,56; *ρ* = −0,097) keine signifikante inverse Korrelation zwischen der Anzahl der richtigen Antworten und der Zeit seit der letzten Fortbildung mehr. Unter den NA konnte keine signifikante Korrelation festgestellt werden (*p* = 0,169; *ρ* = −0,159). Abb. 3 zeigt die prozentualen Werte aller Gruppen.

## Diskussion

Im Rahmen der durchgeführten EKG-Befragung im Single-Choice-Format sollten die Teilnehmenden das EKG-Muster detektieren und keine umfassende eigenständige EKG-Interpretation mit Bestimmung des Lagetyps, Rhythmik, Überprüfung der PQ-Zeit etc. durchführen. Hierbei konnte ein Wissensdefizit in der EKG-Interpretation bei Rettungskräften identifiziert werden. Insgesamt schätzten 63,3 % der Teilnehmenden die 9 Single-Choice-EKG (*n* = 677) korrekt ein (NFS: 61,4 %; NA: 76,5 %). Schwierigkeiten (< 76 % richtige Antworten) bestanden insbesondere bei der Interpretation von AV-Blöcken, verschiedenen Arten von Tachykardien, Vorhofflimmern, Schenkelblöcken, Schrittmacher-EKG und bei der Lagetypbestimmung. Diverse Publikationen haben ebenfalls Wissensdefizite bzw. Verbesserungspotenzial bei der EKG-Interpretation durch NFS und NA festgestellt. In der Untersuchung von Mencl et al. [18] betrug die Sensitivität in der STEMI-Erkennung unter Paramedics 75 %. Die Prozentzahl der korrekten Antwortquote variierte sehr weit, je nachdem wo der Myokardinfarkt vorlag. Der inferiore Myokardinfarkt wurde zu 96 % und der anteriore Myokardinfarkt zu 78 % erkannt, allerdings wurde der laterale Myokardinfarkt nur in 51 % der Fälle erkannt. Nur 39 % der Befragten konnten alle 3 STEMI-EKG korrekt identifizieren und lediglich 3 % interpretierten alle vorgelegten EKG korrekt. In einer weiteren Studie von Wyld, Clegg und Griffin [22], die ebenfalls die STEMI-Erkennung unter Paramedics analysierte, betrug die Sensitivität der STEMI-Erkennung nur 63 %. Darüber hinaus analysierte auch der Review von Funder et al. [8] die STEMI-Erkennung von Paramedics. Hier variierte die STEMI-Erkennung je nach Studie zwischen 58 und 99 %. Ohlow et al. [19] betrachteten die STEMI-Identifizierung von NA. In dieser Studie erkannten 83 % der NA den STEMI. Allerdings wurden andere EKG in nur 30 % der Fälle richtig eingeschätzt. Bei Breckwoldt et al. [4] befundeten Anästhesist*innen zu 84 % und Kardiolog*innen zu 94 % den STEMI korrekt. In der vorliegenden Untersuchung erlangten die Teilnehmenden ähnliche Ergebnisse wie bei Breckwoldt et al., Mencl et al. und Ohlow et al. [4, 18, 19] mit 78,1 % (NFS: 78,1 %; NA: 86,7 %) richtigen STEMI-Einschätzungen. Auch in einer Metaanalyse von Cook et al. [5] traten in allen Ausbildungsebenen unter Medizinstudent*innen und Ärzt*innen Defizite im Bereich der EKG-Interpretation auf. Die Quote der richtigen Antworten lag im Median bei 54 %. Nach einer Fortbildung konnten im Median mit 67 % bessere Ergebnisse erreicht werden. Darüber hinaus wurden auch mit steigendem Ausbildungsstand bessere Ergebnisse erzielt (Medizinstudent*innen 42 %, Assistenzärzt*innen 55,8 %, Fachärzt*innen 68,5 %, Kardiolog*innen/Ärzt*innen in kardiologischer Weiterbildung 74,9 %). Die randomisiert kontrollierte Studie von Kashou [16] untersuchte, wie sich webbasierte EKG-Trainingsprogramme und lehrpersonenbasierter EKG-Unterricht sowie die Anwendung von beidem auf die EKG-Kompetenz von verschiedenen Berufen im Gesundheitswesen (*n* = 1206; Ärzt*innen, Krankenpflegepersonal, Medizinstudent*innen und weitere Gesundheitsfachberufe) auswirkt. Zudem wurden Proband*innen in eine Kontrollgruppe eingeteilt. Alle Interventionsgruppen erreichten eine signifikante Verbesserung nach der jeweiligen Intervention. Teilnehmende der webbasierten Trainingsprogramme erreichten eine Verbesserung von 11,4 % (95 %-KI 9,1; 13,7; *p* < 0,01), Proband*innen des lehrpersonenbasierten Unterrichts 9,8 % (95 % KI 7,8; 11,9; *p* < 0,01) und bei der Anwendung von beidem 11,0 % (95 %-KI 9,2; 12,9; *p* < 0,01). Die Kontrollgruppe zeigte keine signifikante Verbesserung mit 0,8 % (95 %-KI −1,2; 2,8; *p* = 0,54).

Aufgrund der fehlenden diagnostischen Fähigkeiten bei der Befundung von 12-Kanal-EKG sollten dringend die EKG-Aus- und -Fortbildungsprogramme überarbeitet werden. Fortbildungskonzepte sollten die Analysierung des Ist-Stands der EKG-Interpretationskompetenz in Form eines Prätests, das eigentliche Training und Kontrollen durch einen Posttest sowie ein Feedback und wiederkehrende EKG-Fortbildungen umfassen [18]. Zusätzlich könnte das Angebot an Onlinefortbildungen, die Möglichkeit der Nutzung von Telemedizin oder das EKG-Feedback von Kardiolog*innen sowie das Zurverfügungstellen von EKG-Literatur das Angebot zur Verbesserung der EKG-Interpretationskompetenzen abrunden [1, 6, 7, 19, 21]. Es ist zu empfehlen, mindestens eine jährliche EKG-Fortbildung im Rahmen der Pflichtfortbildung für NFS und NA anzubieten, da dies die Handlungssicherheit sowie die subjektive Sicherheit bei der EKG-Beurteilung stärkt, zumal nach einem Jahr das Fortbildungswissen nachzulassen scheint [11, 12]. In der Untersuchung von Henry et al., [12], die die Verbesserung der Handlungssicherheit bezüglich EKG durch die jährliche rettungsdienstliche Pflichtfortbildung inklusive nachfolgender Kompetenzchecks überprüfte, gaben 66,1 % der Befragten an, sich nach den Überprüfungen mäßig sicherer und 23,7 % sich deutlich sicherer in der EKG-Interpretation zu fühlen. Allerdings werden weitere Studien benötigt, um einen genauen Zeitrahmen für eine adäquate EKG-Fortbildung zu definieren. Abschließend muss betont werden, dass zur Erreichung exzellenter prähospitaler Notfallmedizin, die aktiv zur Sicherheit der Patient*innen beiträgt, hochwertige sowie regelmäßige Fortbildungen benötigt werden [9].

## Limitationen

Diese Arbeit hat mehrere Limitationen. Erstens entspricht die Stichprobengröße der Studie von 908 Teilnehmenden einer repräsentativen Größe, jedoch wurde die Stichprobe nicht zufällig gezogen, sodass in der Konsequenz eine Übertragbarkeit auf alle NFS und NA in Deutschland fraglich ist. Zweitens waren einige der nur schwach signifikanten *p*-Werte nach der Bonferroni-Korrektur nicht mehr signifikant. Drittens stellt die hohe Abbruchrate von 231 Teilnehmer*innen, die die Befragung nach der Beantwortung des 1. EKG im Laufe bis zum 9. EKG abgebrochen haben, eine Limitation dar und es ist unklar, warum die Befragten die Befragung abgebrochen haben. Viertens ist es möglich, dass vermehrt Teilnehmende an der Studie teilgenommen haben, die bei dem Thema EKG der Meinung sind, einen guten Wissensstand zu haben (Selektionsbias). Durch diesen Effekt könnten sich in der Befragung im Vergleich zu dem tatsächlichen Wissensstand aller NFS und NA bessere Ergebnisse gezeigt haben. Weiterhin erfolgte die Auswahl der EKG basierend auf der aktuellen Literatur und nur wenige EKG-Bilder gingen zur Befundung in die Umfrage ein. Hier kann nicht ausgeschlossen werden, dass wichtige Bilder fehlen oder die gewählten Darstellungen besonders einfach oder besonders anspruchsvoll waren. Damit gewährt die Arbeit nur einen Einblick in die EKG-Befundung, kann diese aber nicht vollumfänglich darstellen. Dennoch stellt diese Studie einen wichtigen Diskussionsbeitrag bezüglich EKG-Interpretationskompetenz im Rettungsdienst dar und sollte als möglicher Anstoß zur weiteren Forschung in diesem Themenfeld gesehen werden.

## Fazit für die Praxis

- Umfassende Kenntnisse der korrekten EKG-Anlage sowie die Fähigkeit, diese sicher zu interpretieren, ist für eine Tätigkeit im Rettungsdienst unabdingbar.
- Ungeachtet dessen zeigt die vorliegende deutschlandweite Befragung der NFS und NA Defizite auf, da nur 63,3 % der EKG richtig befundet wurden.
- Dementsprechend ist zu empfehlen, das Aus- und Fortbildungskonzept zu überarbeiten und EKG-spezifische Inhalte in die jährlichen Fortbildungen für NFS und NA zu integrieren.
- Zusätzlich könnte ein Angebot an Onlinefortbildungen sowie das Zurverfügungstellen von EKG-Literatur auf den Rettungswachen die EKG-Interpretationskompetenzen verbessern.
- Darüber hinaus besitzt die Telemedizin mittlerweile eine bedeutende Rolle im Rettungsdienst. So bietet die Telemedizin die Möglichkeit schwierige EKG gemeinsam mit Fachexpert*innen zu interpretieren.

## Supplementary Information

Im Onlinesupplement sind die in die Bewertung eingeflossenen EKG mit den Antwortoptionen einzusehen.

## Data Availability

Auf begründete Nachfrage stellen die Autor*innen die Daten zur Umfrage gerne zur Verfügung.
