# Supplementary material for: ECG interpretation in rescue and emergency medical services in Germany: results of a cross-sectional study
Source: Med Klin Intensivmed Notfmed. 2025 Feb 25;121(4):302–9. [Article in German] doi: 10.1007/s00063-025-01252-1 (PMC13132920; doi:10.1007/s00063-025-01252-1)
Supplement: Supplementary file 1 — Im Onlinesupplement sind die in die Bewertung eingeflossenen EKG mit den Antwortoptionen einzusehen. [file 63_2025_1252_MOESM1_ESM.pdf]

13. Bitte beurteilen Sie folgendes EKG. (Zur Information die Herzfrequenz liegt bei 69).

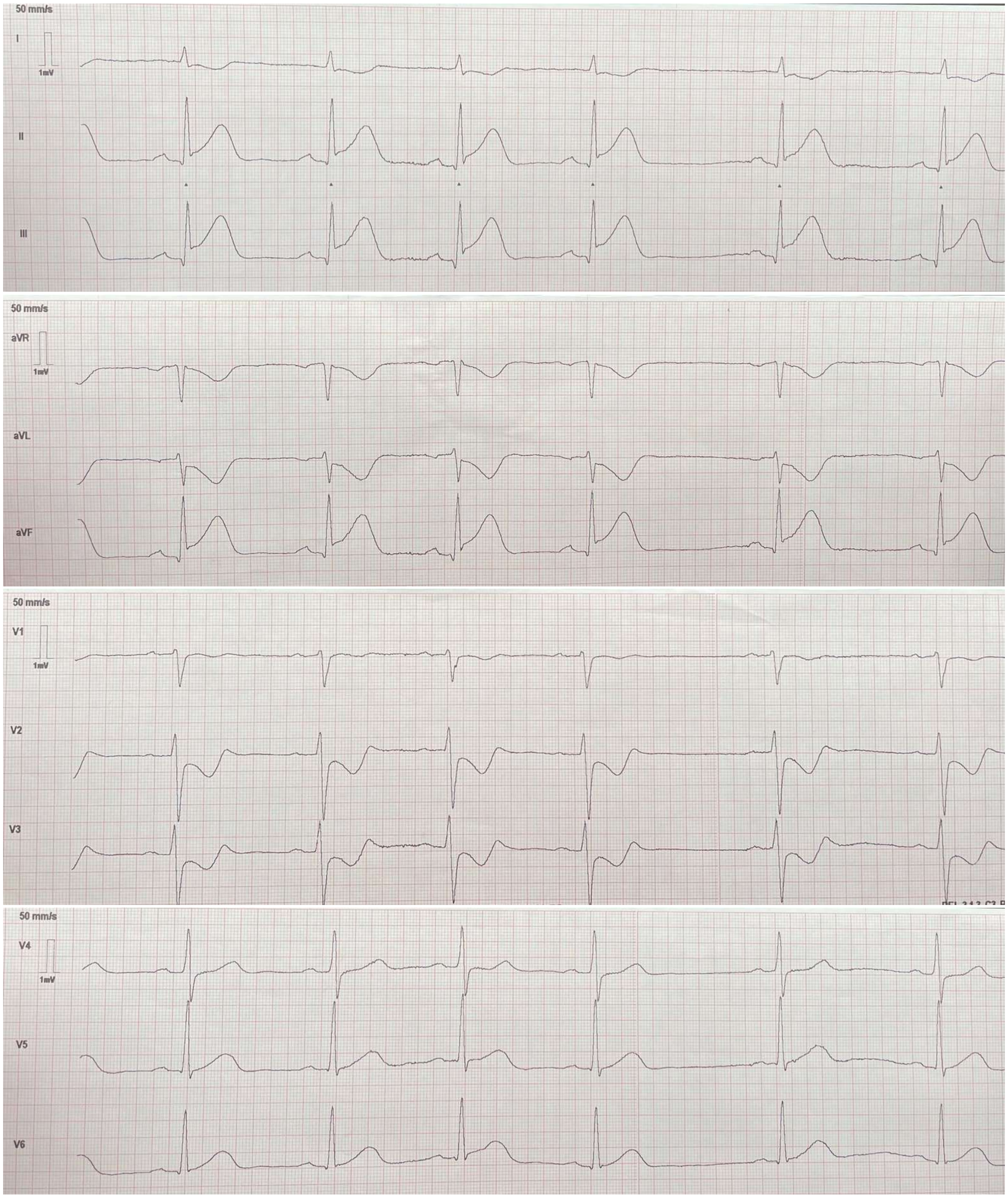

Wählen Sie eine der Antwortmöglichkeiten.

- ○ EKG mit STEMI in der Hinterwand
- ○ EKG mit STEMI in der Vorderwand
- ○ EKG mit NSTEMI
- ○ unauffälliger Sinusrhythmus, ohne Erregungsrückbildungsstörungen
- ○ Sinusrhythmus mit Linksschenkelblock
- ○ Sinusrhythmus mit Rechtsschenkelblock
- ○ Ich kann das EKG nicht beurteilen

14. Bitte beurteilen Sie folgendes EKG. (Zur Information die Herzfrequenz liegt bei 36).

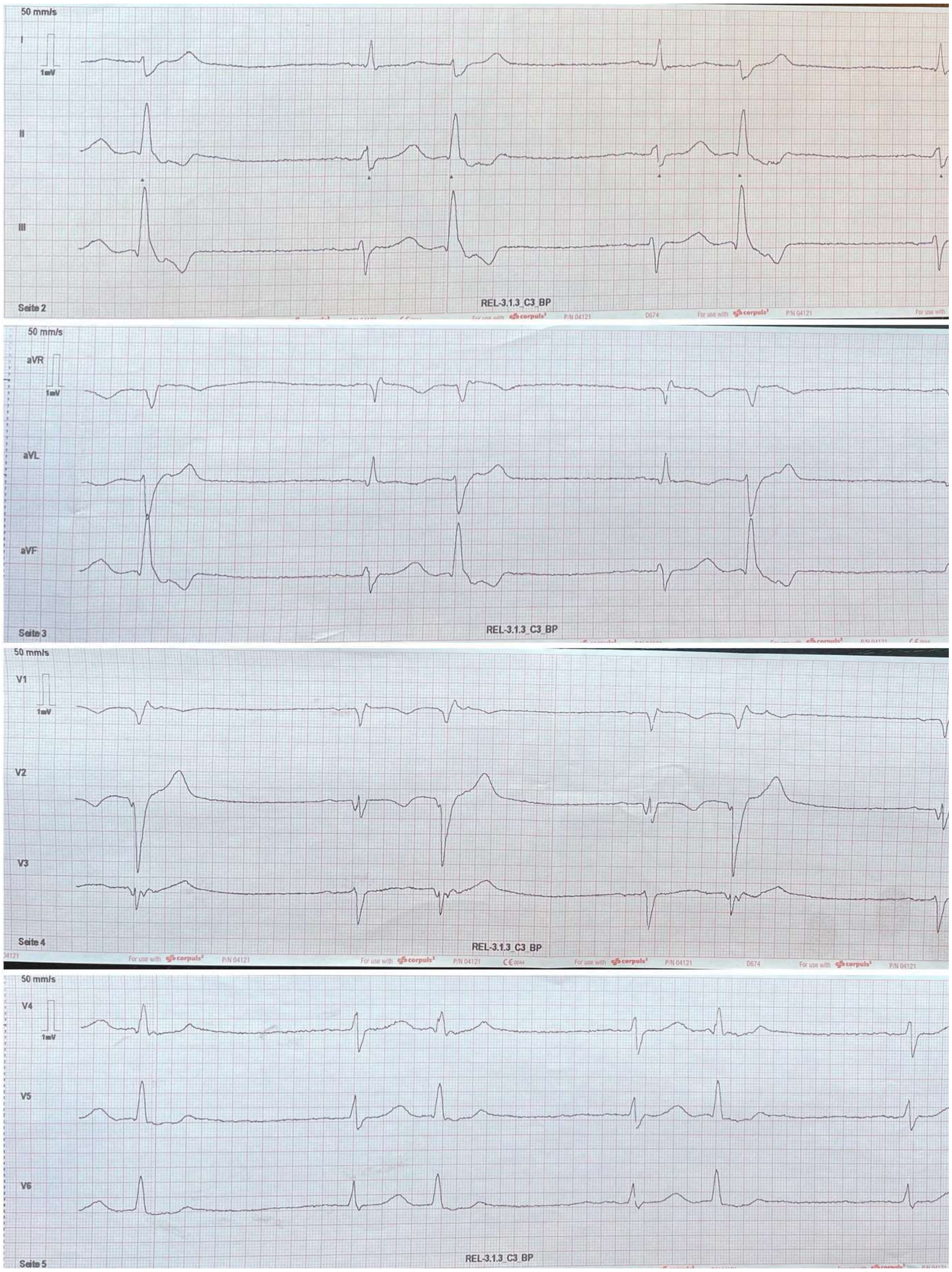

Wählen Sie eine der Antwortmöglichkeiten.

- ○ unauffälliger Sinusrhythmus
- ○ EKG mit Bigeminus
- ○ EKG mit Couplets
- ○ EKG mit Trigeminus
- ○ EKG mit Salven
- ○ Ich kann das EKG nicht beurteilen

15. Bitte beurteilen Sie folgendes EKG. (Zur Information die Herzfrequenz liegt bei 36).

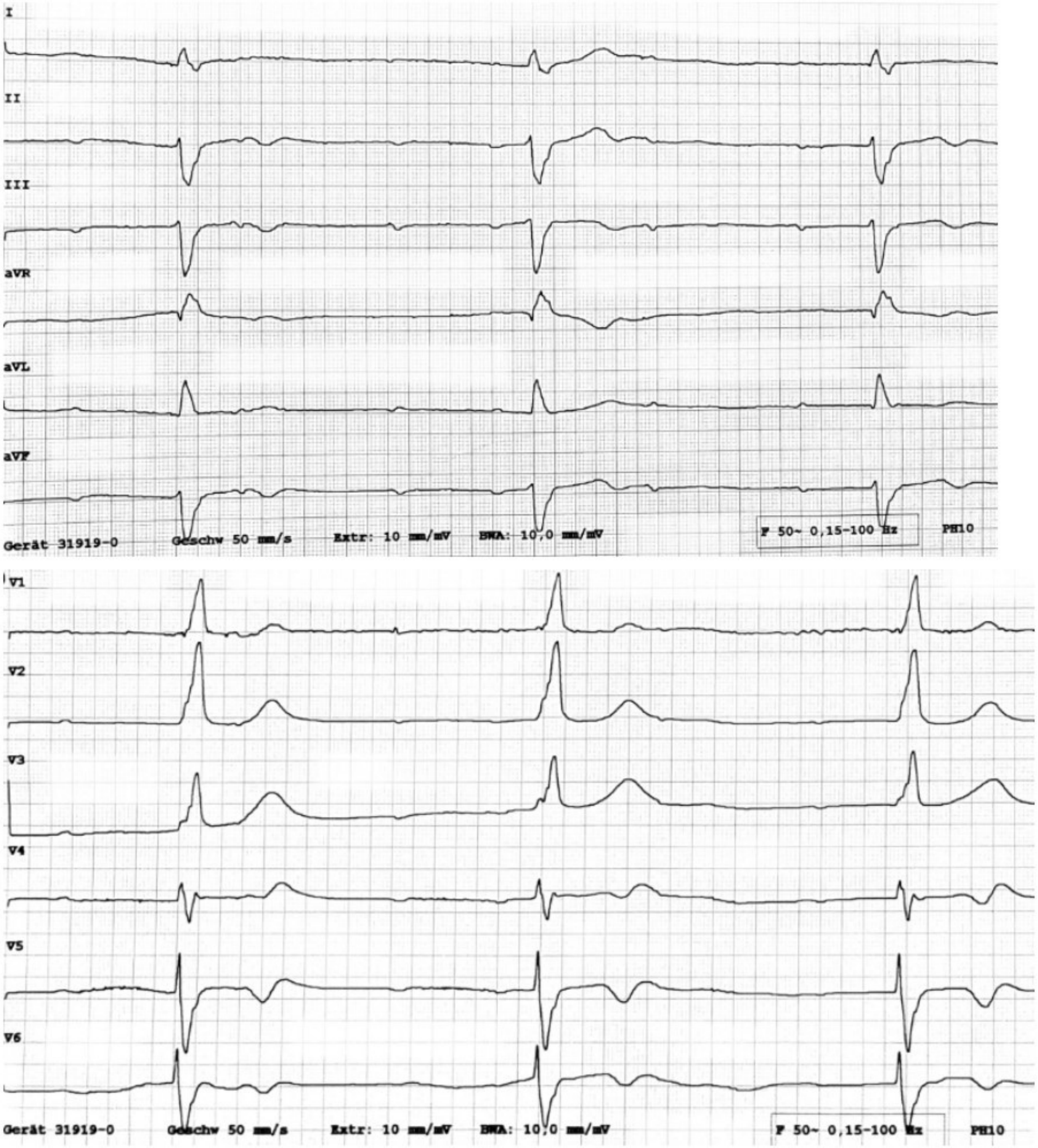

Wählen Sie eine der Antwortmöglichkeiten.

- ○ Sinusbradykardie
- ○ AV-Block 1°
- ○ AV-Block 2° Mobitz I (früher Wenckebach)
- ○ AV-Block 2° Mobitz II (früher Mobitz)
- ○ AV-Block 3°
- ○ Bradyarrhythmia absoluta
- ○ Bradykardes Vorhofflimmern
- ○ unauffälliger Sinusrhythmus
- ○ Ich kann das EKG nicht beurteilen

16. Bitte beurteilen Sie folgendes EKG. (Zur Information die Herzfrequenz liegt bei 194).

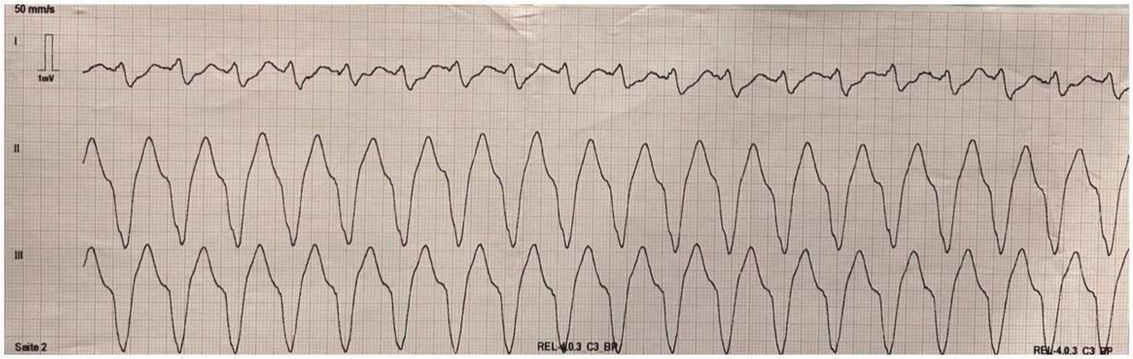

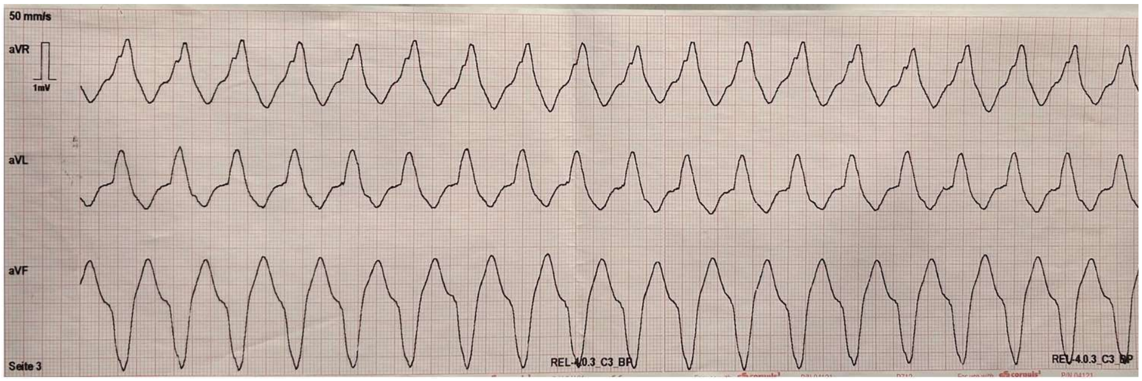

Wählen Sie eine der Antwortmöglichkeiten.

- ○ Supraventrikuläre Tachykardie
- ○ Ventrikuläre Tachykardie
- ○ Kammerflimmern (VF)
- ○ unauffälliger Sinusrhythmus
- ○ EKG mit Bigeminus
- ○ Ich kann das EKG nicht beurteilen

17. Bitte beurteilen Sie folgendes EKG. (Zur Information die Herzfrequenz liegt bei 239.)

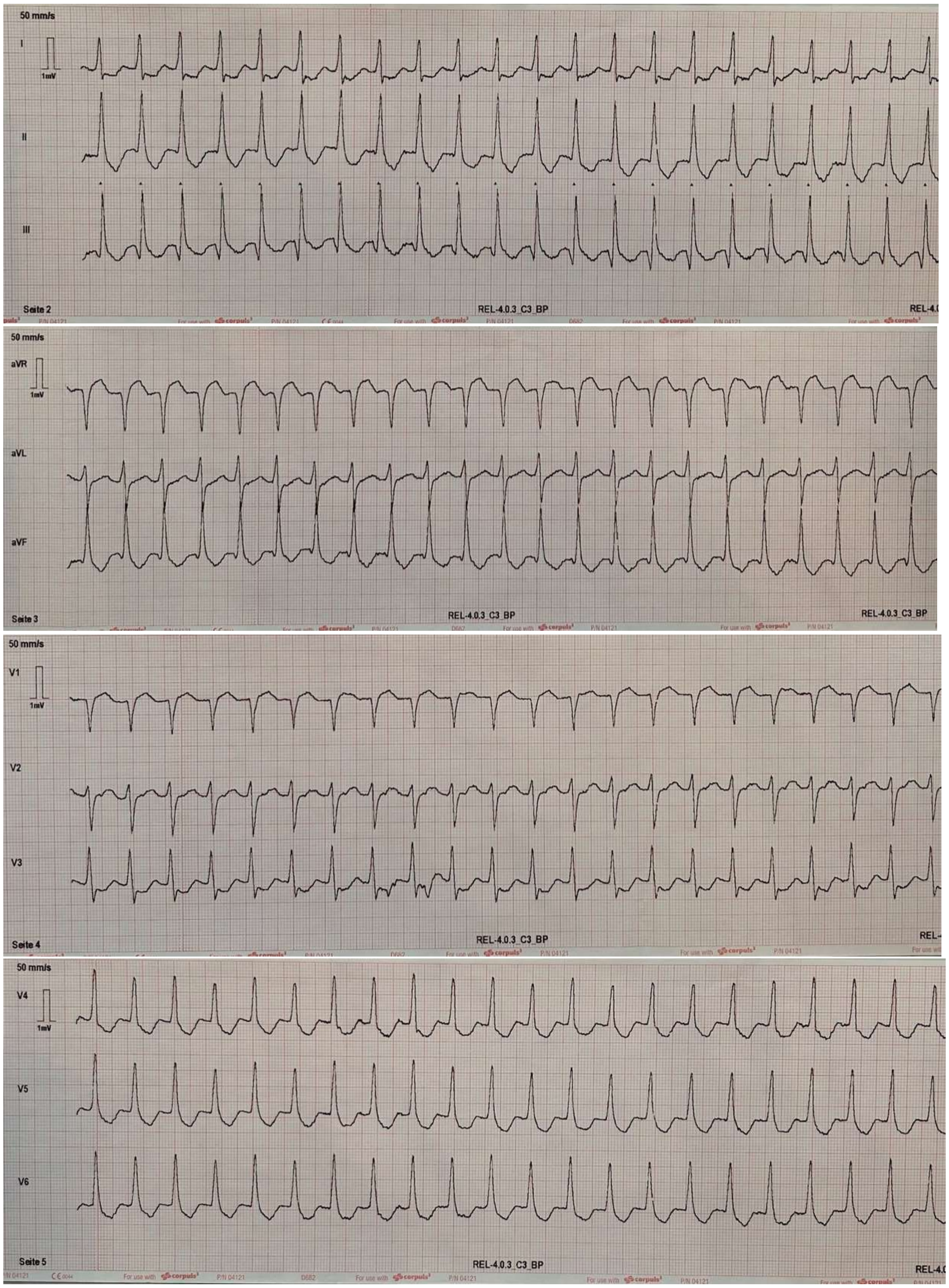

Wählen Sie eine der Antwortmöglichkeiten.

- ○ Supraventrikuläre Tachykardie
- ○ Ventrikuläre Tachykardie
- ○ EKG mit Rechtsschenkelblock
- ○ EKG mit Linksschenkelblock
- ○ AV-Re-Entry-Tachykardie bei Wolff-Parkinson-White (WPW)-Syndrom
- ○ Vorhofflimmern
- ○ Vorhofflattern mit regelmäßiger Überleitung
- ○ Kammerflimmern (VF)
- ○ Sinustachykardie
- ○ Ich kann das EKG nicht beurteilen

18. Bitte beurteilen Sie folgendes EKG. (Zur Information die Herzfrequenz liegt bei 66).

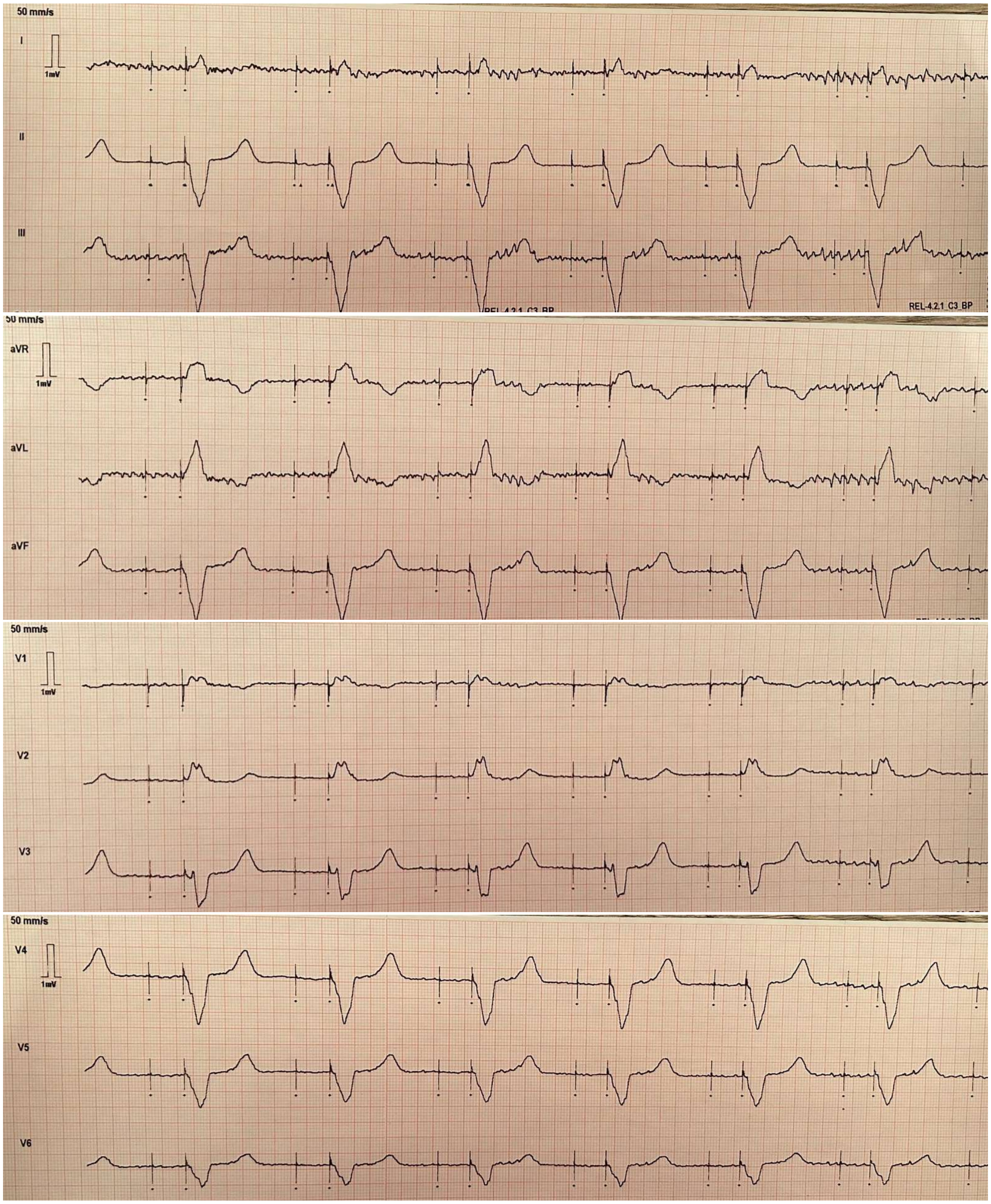

Wählen Sie eine der Antwortmöglichkeiten.

- ○ unauffälliger Sinusrhythmus
- ○ Ventrikuläre Tachykardie
- ○ Supraventrikuläre Tachykardie
- ○ R-auf-T-Phänomen
- ○ Schrittmacher EKG, kann ich nicht näher beurteilen
- ○ Schrittmacher EKG in Betriebsart AAI
- ○ Schrittmacher EKG in Betriebsart VVI
- ○ Schrittmacher EKG in Betriebsart DDD
- ○ Schrittmacher EKG mit Schrittmacherfehlfunktion
- ○ Ich kann das EKG nicht näher beurteilen

19. Bitte beurteilen Sie folgendes EKG. (Zur Information die Herzfrequenz liegt bei 170).

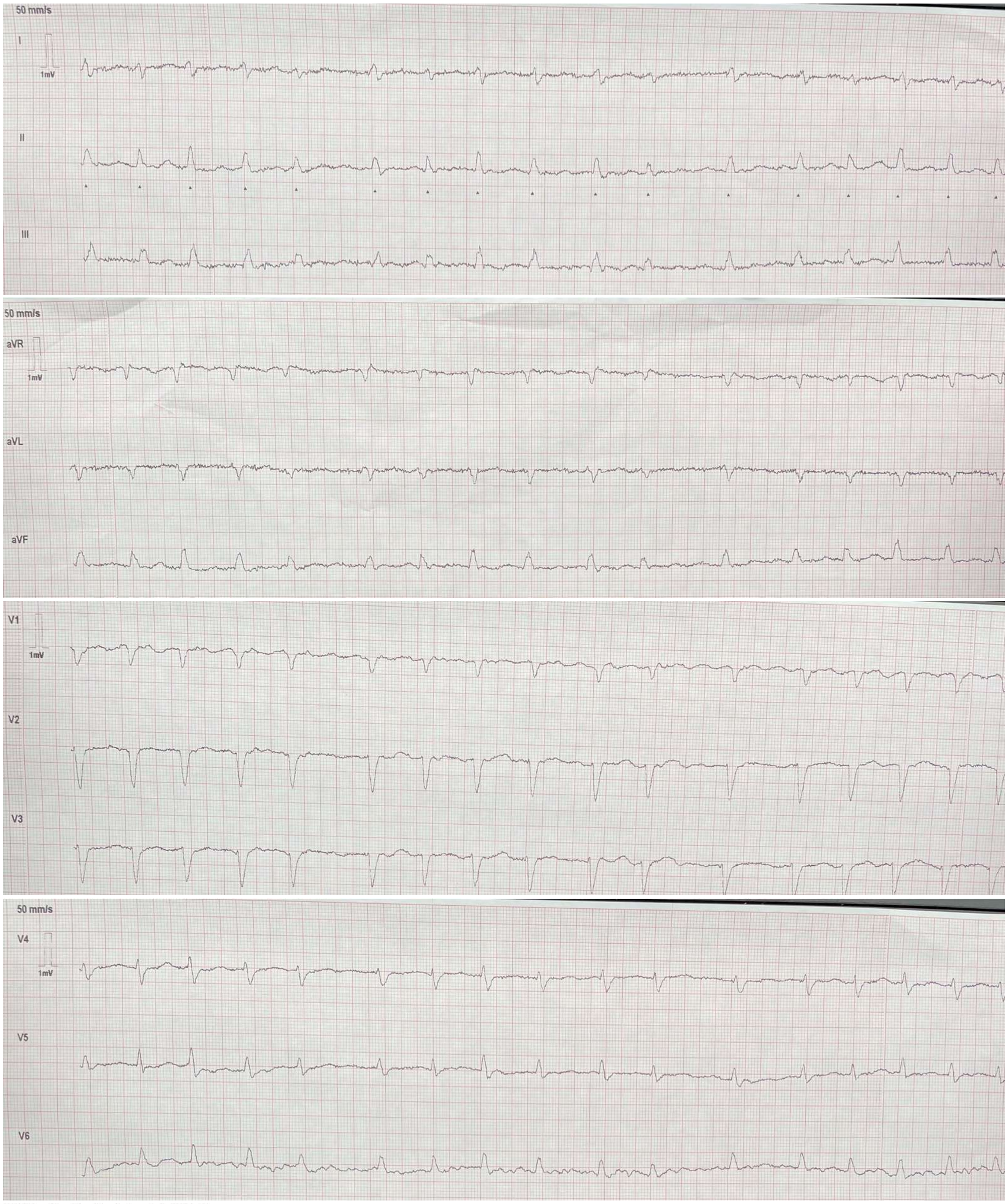

Wählen Sie eine der Antwortmöglichkeiten.

- ○ Supraventrikuläre Tachykardie
- ○ Ventrikuläre Tachykardie
- ○ EKG mit Rechtsschenkelblock
- ○ EKG mit Linksschenkelblock
- ○ AV-Re-Entry-Tachykardie bei Wolff-Parkinson-White- (WPW)-Syndrom
- ○ Tachyarrhythmia absoluta
- ○ Kammerflimmern (VF)
- ○ Sinustachykardie
- ○ Ich kann das EKG nicht beurteilen

20. Bitte beurteilen Sie folgendes EKG. (Zur Information die Herzfrequenz liegt bei 79).

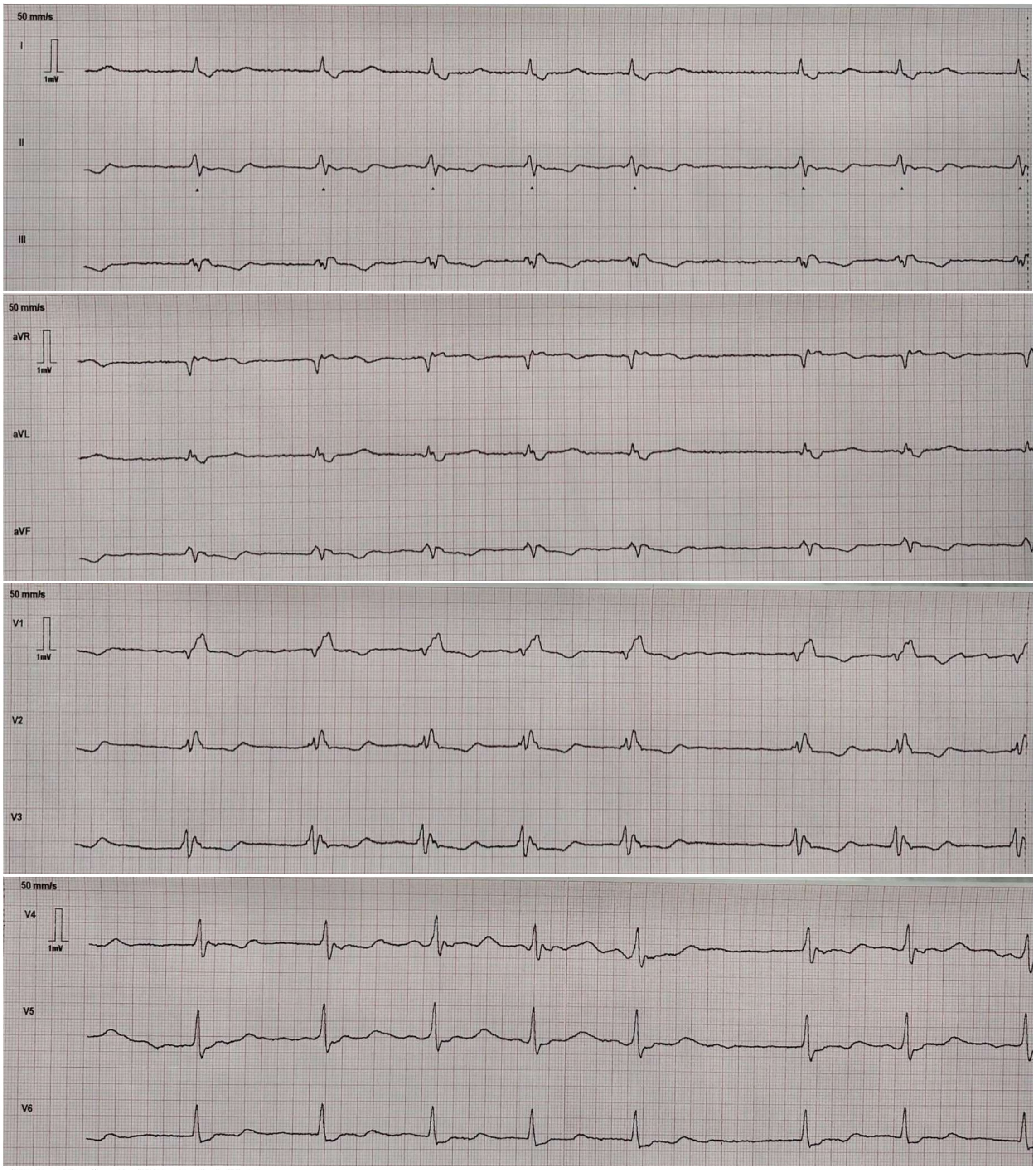

Wählen Sie eine der Antwortmöglichkeiten.

- ○ EKG mit STEMI der Hinterwand
- ○ EKG mit STEMI der Vorderwand
- ○ EKG mit NSTEMI
- ○ unauffälliger Sinusrhythmus
- ○ Sinusrhythmus mit Linksschenkelblock
- ○ Sinusrhythmus mit Rechtsschenkelblock
- ○ Vorhofflimmern mit Linksschenkelblock
- ○ Vorhofflimmern mit Rechtsschenkelblock
- ○ Ich kann das EKG nicht beurteilen

21. Welcher Lagetyp liegt im folgenden EKG vor?

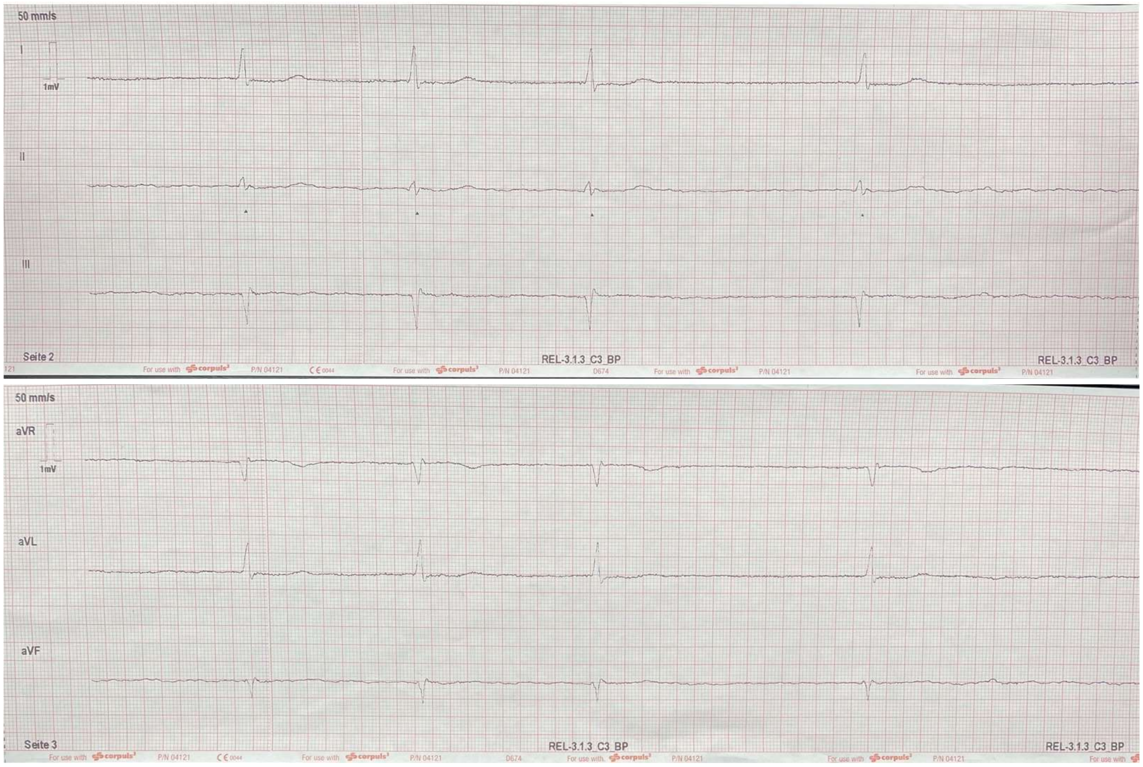

Wählen Sie eine der Antwortmöglichkeiten.

- ○ Indifferenztyp
- ○ Steiltyp
- ○ Rechtstyp
- ○ Überdrehter Rechtstyp
- ○ Linkstyp
- ○ Überdrehter Linkstyp
- ○ S1Q3-Typ
- ○ Ich kann den Lagetyp nicht bestimmen
